# Heparanase‐induced endothelial glycocalyx degradation exacerbates lung ischemia/reperfusion injury in male mice

**DOI:** 10.14814/phy2.70113

**Published:** 2024-10-24

**Authors:** Kentaro Noda, Neha Atale, Amer Al‐Zahrani, Masashi Furukawa, Mark E. Snyder, Xi Ren, Pablo G. Sanchez

**Affiliations:** ^1^ Division of Lung Transplant and Lung Failure, Department of Cardiothoracic Surgery University of Pittsburgh Pittsburgh Pennsylvania USA; ^2^ Division of Pulmonary, Allergy, and Critical Care Medicine, Department of Medicine University of Pittsburgh Pittsburgh Pennsylvania USA; ^3^ Department of Biomedical Engineering Carnegie Mellon University Pittsburgh Pennsylvania USA; ^4^ Section of Thoracic Surgery, Department of Surgery University of Chicago Chicago Illinois USA

**Keywords:** endothelial glycocalyx, endothelial protection, heparanase, lung ischemia reperfusion injury, matrix metalloproteinase

## Abstract

The endothelial glycocalyx (eGC) is a carbohydrate‐rich layer on the vascular endothelium, and its damage can lead to endothelial and organ dysfunction. Heparanase (HPSE) degrades the eGC in response to cellular stress, but its role in organ dysfunction remains unclear. This study investigates HPSE's role in lung ischemia–reperfusion (I/R) injury. A left lung hilar occlusion model was used in B6 wildtype (WT) and HPSE genetic knockout (^−/−^) mice to induce I/R injury in vivo. The left lungs were ischemic for 1 h followed by reperfusion for 4 h prior to investigations of lung function and eGC status. Data were compared between uninjured lungs and I/R‐injured lungs in WT and HPSE^−/−^ mice. WT lungs showed significant functional impairment after I/R injury, whereas HPSE^−/−^ lungs did not. Inhibition or knockout of HPSE prevented eGC damage, inflammation, and cellular migration after I/R injury by reducing matrix metalloproteinase activities. HPSE^−/−^ mice exhibited compensatory regulation of related gene expressions. HPSE facilitates eGC degradation leading to inflammation and impaired lung function after I/R injury. HPSE may be a therapeutic target to attenuate graft damage in lung transplantation.

## 1 INTRODUCTION

Endothelial preservation is important for organ preservation during transplantation. There are many chances to injure or protect the graft endothelium during the transplantation process including donor management, ex vivo graft evaluation, intraoperative patient management and ischemia–reperfusion (I/R). (Coster et al., 2023; Jungraithmayr, 2020; Machuca et al., 2015; Srivastava et al., 2021) Failure to protect the endothelium results in endothelial dysfunction and can lead to organ failure after transplantation. In lung transplantation, primary graft dysfunction (PGD), which is defined as graft edema and poor gas exchange function that occurs within 72 h of transplantation, is an acute phenotype of endothelial dysfunction. (Cantu et al., 2022) PGD is the primary cause of early posttransplant mortality, and unfortunately, clinical strategies to prevent PGD are currently lacking. Identifying clinically applicable, practical, therapeutic strategies to preserve endothelial cells in grafts may reduce the incidence of PGD and improve posttransplant outcomes.

The endothelial glycocalyx layer is a carbohydrate‐rich matrix of proteoglycans, glycosaminoglycans (GAGs), and soluble proteins covering the luminal surface of endothelial cells that protect them and support their functions. (Uchimido et al., 2019) The endothelial glycocalyx is sensitive to cellular stress. Degradation of the endothelial glycocalyx results in endothelial dysfunction and is associated with acute failure in many organs and some chronic disease states. (Reitsma et al., 2007) Very recently, research efforts revealed that maintaining the integrity of the endothelial glycocalyx may contribute to protecting lung grafts through better endothelial preservation. (Jungraithmayr, 2020) Our group investigated the role of the endothelial glycocalyx during organ transplantation and found a clinical association between poor graft quality during ex vivo lung perfusion and increased glycocalyx components in perfusate. (Noda et al., 2023) We also demonstrated that glycocalyx degradation in lung grafts, induced by in vivo warm ischemia or ex vivo lung perfusion, was associated with edema and poorer posttransplant graft function in a small animal model. (Noda et al., 2021, 2023) Sladden and colleagues previously reported that the lung donor plasma levels of syndecan‐1 and hyaluronan, components of the endothelial glycocalyx, were highly associated with graft utilization and the incidence of severe PGD. (Sladden et al., 2019) These studies suggest that the status of the glycocalyx may be directly linked to graft quality and that protection of the glycocalyx may lead to improved endothelial protection, better preservation of graft quality, and improved posttransplant outcomes. In current practice, there are no therapeutic targets for endothelial preservation, and no feasible endothelial preservation strategies have been applied throughout the lung transplant process.

Heparanase‐1 (HPSE), an endo‐β‐D‐glucuronidase responsible for degrading the extracellular matrix, may be a key regulator of glycocalyx integrity. (Vlodavsky et al., 2020) In response to cellular stress, HPSE is cleaved by the cathepsins to its 55‐kDa of active form, which is then released to the extracellular space and specifically degrades heparan sulfate chains on the cell‐surface glycoproteins. Several HPSE inhibitors have been identified and are being tested for their ability to inhibit cancer metastasis by inhibiting the breaking down of the extracellular matrix. (Mohan et al., 2019; Sanderson et al., 2017) In kidney disease, HPSE is known to induce endothelial dysfunction leading to acute organ failure through its ability to catalyze shedding of the endothelial glycocalyx. (Rabelink et al., 2017) HPSE and cleaved heparan sulfate are associated with illness severity in critically ill patients with sepsis. (Schmidt et al., 2014) Our group demonstrated that the HPSE inhibition preserved the endothelial glycocalyx in lung grafts during warm ischemic insult or ex‐vivo lung perfusion, leading to better lung graft quality after transplantation in rats. (Noda et al., 2021, 2023) In this study, we aimed to investigate the role of HPSE in glycocalyx dynamics in response to I/R insults typically encountered during the lung transplant process using mouse models of lung I/R injury, an HPSE inhibitor, and *HPSE*‐knockout mice (*HPSE* ^−/−^).

## 2 MATERIALS AND METHODS

### 2.1 Study design

We generated lung I/R injury to the left lung in vivo using male C57BL/6J wild‐type (WT) and *HPSE* ^−/−^ mice. We randomly assigned WT mice into three groups (1) WT no injury; (2) WT I/R injury, in which the left lung was subjected to 1 h of ischemia and 4 h of reperfusion; and (3) WT I/R SF4, in which the mice were pretreated with an iminosugar‐based HPSE inhibitor, SF4 (heparastatin SF4; DiagnoCine LLC, Hackensack, NJ), prior to 1 h of ischemia and 4 h of reperfusion. We randomly assigned *HPSE* ^−/−^ mice into two groups (1) *HPSE* ^−/−^ uninjured and (2) *HPSE* ^−/−^ I/R injury, in which the left lung was subjected to 1 h of ischemia and 4 h of reperfusion. The samples of lung tissue and blood plasma were collected and analyzed to assess I/R injury, focusing on lung function, graft quality, endothelial dysfunction, status of the glycocalyx and glycocalyx‐degrading enzymes, and immune cell infiltration into the lungs (Supplemental Methods—Data S1).

### 2.2 Animals and Ethical Approval

C57BL/6J WT mice were purchased from the Jackson Laboratory (Bar Harbor, ME). Animals were maintained in laminar flow cages in a specific pathogen‐free animal facility at the University of Pittsburgh and given a standard diet (Prolab® IsoPro® RMH 3000; LabDiet, St. Louis, MO) and water ad libitum. All procedures were approved by the Institutional Animal Care and Use Committee (IACUC) at the University of Pittsburgh (Protocol# 22081818) and performed according to the guidelines of the IACUC and the National Research Council's Guide for the Humane Care and Use of Laboratory Animals.

### 2.3 Heparanase‐1 knockout mice model

**FIGURE 1:**
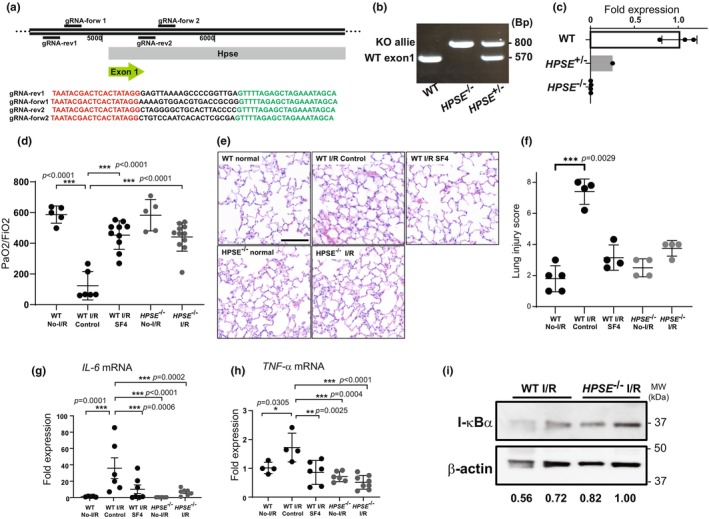
The effect of heparanase‐1 (HPSE) on lung ischemia–reperfusion (I/R) injury in wild‐type (WT) and *HPSE* ^−/−^ mice. (a) The strategy used to develop *HPSE* ^−/−^ mice using Crispr‐Cas9 and gRNA sequences. (b) PCR genotyping and (c) real‐time PCR quantitation of mRNA expression for the *HPSE* gene in the lungs of WT, *HPSE* ^+/−^, and *HPSE* ^−/−^ mice. The left lungs after 1 h of ischemia followed by 4 h of reperfusion were evaluated for injury based on (d) PaO2/FiO2, (e, f) H&E staining/injury score, mRNA expression levels for proinflammatory cytokines (g) interleukin‐6 (IL‐6) and (h) tumor necrosis factor‐α (TNF‐α), and (i) western blotting for inhibitor of nuclear factor κB α (ΙκBα) expression. *p < 0.05, **p < 0.01, ***p < 0.001. WT No‐I/R, uninjured lungs from wild‐type mice; WT I/R control, lungs from wild type mice after 1 h ischemia/4 h reperfusion; WT I/R SF4, lungs from wild type mice pretreated with HPSE inhibitor followed by 1 h ischemia/4 h reperfusion; *HPSE* ^−/−^ No‐I/R, uninjured lungs from *HPSE* ^−/−^ mice; *HPSE* ^−/−^ I/R, lungs from *HPSE* ^−/−^ mice after 1 h ischemia/4 h reperfusion.

*HPSE* ^−/−^ mice were generated by the Innovative Technologies Development (ITD) core at the University of Pittsburgh. *HPSE* ^−/−^ mice have been generated by others previously. (Poon et al., 2014; Zcharia et al., 2009) Previously reported strategies for *HPSE‐1* gene deletion commonly targeted the promoter region of exon 1; thus, we employed a similar strategy using the Crispr‐Cas9 system (Figure 1a). Briefly, a pair of target sequences were introduced to allow 2 double‐strand breaks by the Cas9 nuclease. This created a deletion of the intervening segment by non‐homologous end joining. (Hall et al., 2018) The pronuclei of fertilized embryos (C57BL/6J) produced by natural mating were microinjected with a mixture of 1.3 μM EnGen Cas9 protein (New England Biolabs, Cat.No.M0646T), and designed Cas9 gRNAs (sequences are listed in Table 1), each at 21 ng/μL. The embryos that developed to the 2‐cell stage were transferred to the oviducts of CD1 female surrogate mice. The potential founder mice were genotyped, and their DNA was sequenced to identify mice with the correct sequence of the knockout allele for breeding. The potential knockout founders were backcrossed with WT C57BL/6J mice to establish the colony on the C57BL/6J background.

**TABLE 1:** Sequences for gRNA and PCR primers for genotyping.

Target	Sequence
gRNA‐Rev1	TAATACGACTCACTATAGGGAGTTAAAAGCCCCGGTTGAGTTTTAGAGCTAGAAATAGCA
gRNA‐Ford1	TAATACGACTCACTATAGGAAAAGTGGACGTGACCGCGGGTTTTAGAGCTAGAAATAGCA
gRNA‐Rev2	TAATACGACTCACTATAGGCTAGGGGCTGCACTTACCCCGTTTTAGAGCTAGAAATAGCA
gRNA‐Ford2	TAATACGACTCACTATAGGCTGTCCAATCACACTCGCGAGTTTTAGAGCTAGAAATAGCA
Genotype primer forward 1	CCAAGCCTCAGAGCAACTCTT
Genotype primer forward 2	TGCCCCAGCTCTCCCGGCA
Genotype primer Reverse	ACACCAGCTTACTGGTCTTACA

### 2.4 Mice ischemia reperfusion injury model

A hilar ligation model of lung I/R was performed as described previously. (Huerter et al., 2016; Kumar et al., 2020) The left lung was occluded using 6–0 surgical polypropylene suture and subjected to 1 h of ischemia followed by 4 h of reperfusion. In the WT I/R SF4 group, 50 μL of 40 μM SF4 was administered by intraperitoneal injection 1 h prior to occlusion (clamping) to generate ischemia. After 4 h of reperfusion, animals were anesthetized and subjected to mechanical ventilation. The lung function was evaluated as written below and the blood was sampled with 20 μL of 1 M EDTA for further analyses then euthanized by exsanguination. Tissue sampling was performed after euthanasia.

### 2.5 Assessment of lung function

The lung function after I/R was determined by measuring the P/F ratio (partial pressure of oxygen in arterial blood [PaO_2_]/ fraction of inspiratory oxygen [FiO_2_]). A ventilator was used to deliver 100% O_2_ and then the right lung was clamped. After 5 min of single lung ventilation, blood was sampled from the left ventricle and analyzed by a blood gas analyzer (iSTAT, Abbott Laboratories, Chicago, IL).

### 2.6 In vitro β‐D‐glucuronidase activity assay

HPSE activity was measured with a β‐Glucuronidase activity assay using 4‐methylumbelliferyl‐β‐D‐glucuronide (4‐MUG) substrate (Millipore‐Sigma, Burlington, MA). (Noda et al., 2023) In brief, tissue lysate was incubated with 10 μM 4‐MUG at pH 4 in PBS at 37°C. Fluorescence (*λ*ex 360 nm/*λ*em 460 nm) was continuously measured for 60 min using a fluorescence microplate reader (BioTech Synergy HTX, Agilent Technologies, Santa Clara, CA).

### 2.7 In vivo vascular permeability assay

Vascular permeability was measured using the Evans Blue dye (EBD) method. (Kumar et al., 2020) Briefly, EBD was injected through the jugular vein after reperfusion. Following completion of the I/R experiment, excess EBD was removed by perfusion of phosphate buffered saline (PBS) via the pulmonary artery. The EBD remaining in lung tissue was extracted with formamide and quantitated by measuring the absorbance of the extractants at 620 nm. The values were normalized to tissue weight.

### 2.8 1,9‐dimethylmethylene blue (DMMB) assay

The plasma glycosaminoglycan (GAG) levels in mice after I/R injury were quantitated using a 1,9‐dimethylmethylene blue (DMMB) assay as described previously. (Barbosa et al., 2003; Noda et al., 2023) The DMMB solution (pH ~ 3.0) was prepared by dissolving 16 mg/L DMMB in distilled H_2_O containing 3.04 g/L glycine, 1.6 g/L NaCl and 95 mL 0.1 M acetic acid. Briefly, 1 mL DMMB solution was mixed with 70 μL of plasma then incubated for 18 h at room temperature. The tubes were then centrifuged at 14,000 rpm for 20 min at 4°C. Supernatants were carefully decanted, 100 mL dissolvent buffer (4 M guanidine hydrochloride, 50 mM sodium acetate, 10% propan‐1‐ol, pH 6.8) was added to each pellet, and the tubes were incubated at 40°C and vortexed until the GAG‐DMMB pellets were completely dissolved. GAG content in each sample was measured spectrophotometrically at 656 nm.

### 2.9 Real‐time reverse transcriptase PCR

The levels of messenger RNA (mRNA) were assessed by SYBR Green 2‐step, real‐time, reverse transcription‐polymerase chain reaction (RT‐PCR) as previously described (Noda et al., 2012) using the ΔΔCT method. The expression of *β‐actin* mRNA served as an internal control and was used to normalize the mRNA expression of all target genes.

### 2.10 Western blotting

Western blotting was performed using the tissue lysates prepared from the lungs after I/R injury as described previously. (Kumar et al., 2020) Total extracted proteins were resolved by 10% SDS‐PAGE followed by transfer to 0.2 μm nitrocellulose membranes (Bio‐Rad Laboratories, Hercules, CA, USA). Membranes were blocked with 5% non‐fat milk at room temperature for 2 h and then incubated overnight at 4°C with the following primary antibodies: inhibitor of NFκB‐α (IκBα; #9242, Cell Signaling Technology, Inc., Danvers, MA), caveolin‐1 (#3238, Cell Signaling Technology, Inc.) and *β‐actin* (#A1978, Millipore‐Sigma). Membranes were then incubated with either goat anti‐mouse IgG (H + L) (#31430, Thermo Fisher Scientific Inc.; 1:5000) or goat anti‐rabbit IgG (H + L) (#31460, Thermo Fisher Scientific Inc.; 1:3000) horseradish peroxidase‐conjugated polyclonal secondary antibodies at room temperature for 2 h. Proteins were visualized using an enhanced chemiluminescence kit (Abcam), and protein bands were imaged using Image Lab software (Version 6.0; Bio‐Rad Laboratories, Inc., Hercules, CA). The intensities of protein bands were quantitated by Image J software (National Institutes of Health, Bethesda, MD).

### 2.11 Gelatin zymography

To determine the activity of the pro and active forms of matrix metalloprotease (MMP) 2 and MMP9 in lung tissue after I/R injury, gelatin zymography was performed as described previously. (Noda et al., 2021, 2023) The mouse lungs was homogenized in cold PBS, and the homogenate was applied to the gel. After electrophoresis, the gel was stained with Coomassie Brilliant Blue R‐250 (Bio‐Rad) and imaged (ChemiDoc, Bio‐Rad). Enzyme activity, visualized as clear bands against the dark blue background, was quantified using Image‐J software.

### 2.12 MMP14 activity assay

To determine MMP14 activity in the lung tissue, we used a fluorogenic MMP14 substrate (MMP‐14 substrate I, Fluorogenic; Sigma). Lungs were homogenized, and 10 μL of the homogenate was mixed in 200 μL of 50 μM MMP14 substrate in a buffer (50 mM Tris HCl, 10 mM CaCl2, pH 7.5). Fluorescence (*λ*ex 388 nm/*λ*em 400 nm) was continuously measured for 60 min using a fluorescence microplate reader (BioTech Synergy HTX, Agilent Technologies, Santa Clara, CA).

### 2.13 Histopathology and immunofluorescence staining

Formalin‐fixed, paraffin‐embedded (FFPE) lung tissues were sectioned to 4‐μm thickness and stained with hematoxylin and eosin. Acute lung injury was blindly scored according to previously described criteria. (Kawamura et al., 2011, 2013) Briefly, acute lung injury was scored according to (1) thickness of the alveolar wall, (2) infiltration or aggregation of neutrophils in airspace, the alveolar wall, or the vessel wall, and (3) alveolar congestion, and each item was graded on a four‐point scale. Each component ranged from 0 to 3, with higher scores indicating more severe damage. A total lung injury score was calculated as the sum of the three components (from 0 to 9). At least five fields were chosen randomly from each section and were examined at ×400 magnification.

FFPE sections of lung tissue were stained for immunofluorescent imaging using primary antibodies for syndecan‐1 (#sc‐12,765, Santa Cruz) and CD31 (Abcam, Cambridge, UK) and with Hoechst 33342 dye (Thermo Scientific). Donkey Cy3‐conjugated anti‐mouse IgG (#AP192C, MilliporeSigma, Burlington, MA) and Cy5‐conjugated goat anti‐rabbit IgG (#A10523, Thermo Scientific) secondary antibodies were used to detect the primary antibodies. Stained slides were scanned with a whole‐slide image scanner (Axio Scan.Z1; Carl Zeiss AB, Oberkochen, Germany) and analyzed with digital image processing software (ZEN lite blue edition; Carl Zeiss).

### 2.14 Determining wet‐to‐dry (w/d) weight ratio

The lungs were flushed in in‐situ by injecting PBS via pulmonary artery to remove the blood then excised and measured the wet weight of tissue then placed in a 60°C oven to dry for 72 h. Tissues were weighed again after drying to determine the wet‐to‐dry lung weight ratio. (Kawamura et al., 2013).

### 2.15 Flow cytometry

Flow cytometry was performed to define the cell population migrating into the lungs after I/R injury. The lung tissue was enzymatically digested using collagenase type I (Worthington Biochemical Corporation, Lakewood, NJ), and a single cell suspension was obtained after straining using 40 μm filter and lysing of the red blood cells. The cells were stained with trypan blue and counted to confirm their viability >90%. Cells from the suspension (1 × 10^6^ cells per sample) were stained using antibodies for CD3e (clone 500A2, BD, Franklin Lakes, NJ), CD4 (clone GK1.5, BioLegend, San Diego, CA), CD8 (clone 53–6.7, BioLegend), CD11B (clone M1/70, BD), CD11C (clone N148, BioLegend), CD31 (clone 390, BioLegend), CD45 (clone 30‐F11, BD), CD86 (clone A17199A, BioLegend), F4/80 (T45‐2342, BD), Siglec F (clone S17007L, BioLegend), Ly6C (clone HK1.4, BioLegend), and Ly6G (clone 1A8, BioLegend) and isotype control antibodies (BioLegend). After staining, the samples were fixed and then run on a cytometer (LSRFortessa™ Cell Analyzer, BD). The flow cytometry data were analyzed with FCS Express software (De Novo Software, Pasadena, CA).

### 2.16 Statistical analysis

All data were analyzed using SPSS Version 25 statistical software (SPSS Inc., Chicago, IL). Results are expressed as mean ± standard deviation (SD) and graphs were produced by the GraphPad Prism (GraphPad Software, LLC., Boston, MA). The data with multiple groups were analyzed with one‐way analysis of variance followed by post hoc analysis with the Bonferroni correction for multiple comparisons. A probability level of *p* < 0.05 was considered statistically significant.

## 3 RESULTS

### 3.1 Deletion or inhibition of HPSE leads to a higher tolerance for I/R injury

We successfully established *HPSE* ^−/−^ mice using the Crispr‐Cas9 system to delete the promoter region of exon 1 (Figure 1b). We additionally confirmed that *HPSE1* mRNA was undetectable by real‐time PCR (Figure 1c).

After 1 h of ischemia followed by 4 h of reperfusion, the P/F ratio of the left lungs in WT mice was significantly diminished as compared with the P/F ratio in the uninjured lungs of WT mice. In contrast, the P/F ratio did not decrease as markedly after I/R injury in WT mice pretreated with SF4 or in *HPSE* ^−/−^ mice (Figure 1d). Stained histological sections revealed alveolar parenchymal edema in all mice after I/R injury; however, SF4‐treated WT mice and *HPSE* ^−/−^ mice had milder histological changes as compared with the WT mice (Figure 1e). Additionally, the lung injury scores in WT I/R injury group were significantly higher than those of uninjured WT lungs however, no significant difference was found among other groups (Figure 1f). The mRNAs for *IL‐6* and *TNF‐α*, proinflammatory mediators, were significantly upregulated in the lungs of WT control mice after I/R injury as compared with uninjured WT lungs. This upregulation was significantly attenuated in the lungs of SF4‐treated WT mice and *HPSE* ^−/−^ mice (Figure 1g,h). In addition, the expression of inhibitor of nuclear factor κB‐α (IκBα) protein in the lungs was reduced in WT mice after I/R injury but was well preserved in the lungs of *HPSE* ^−/−^ mice after I/R (Figure 1i and Figure S1).

### 3.2 The integrity of the pulmonary endothelium in HPSE ^−/−^ mice was well preserved after I/R injury

**FIGURE 2:**
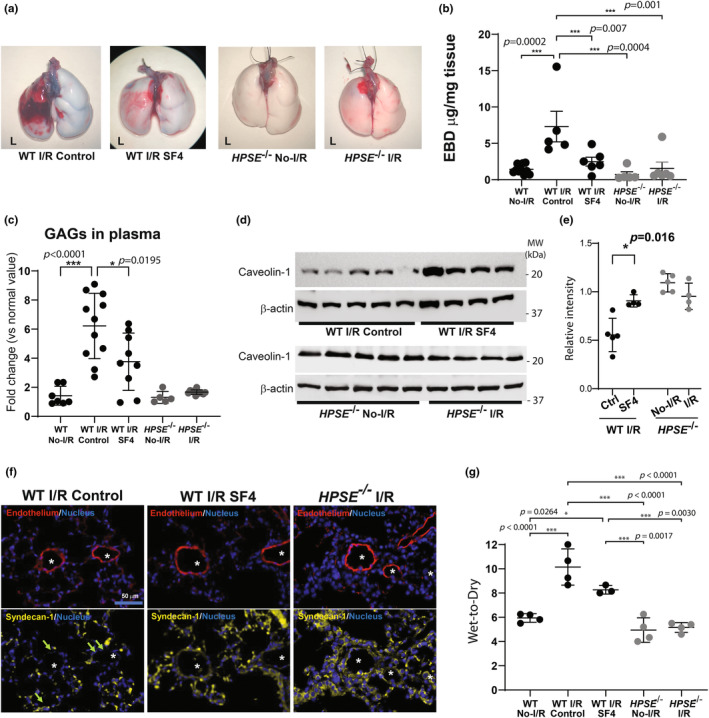
HPSE inhibition or deletion reduces endothelial dysfunction in the lungs after I/R injury. Endothelial barrier function in the lung after ischemia–reperfusion (I/R) injury was evaluated by (a) gross assessment of the lungs after Evans blue dye (EBD) administration and (b) quantitative measurement of EBD extracted from the lung tissue. (c) Plasma glycosaminoglycan (GAG) levels in wild type (WT) and *HPSE* ^−/−^ mice after lung I/R injury were determined using 1,9‐dimethylmethylene blue (DMMB) assay and normalized using the plasma level in uninjured mice of each genotype to calculate fold change. (d, e) Western blotting analysis for caveolin‐1 with *β‐Actin* as an internal control, in lung tissue after 1 h ischemia/4 h reperfusion and their quantitative analysis (cav‐1/ *β‐Actin*) (e). (f) Representative images of the immunofluorescent staining for endothelial cells (CD31), syndecan‐1, and nuclei in lung tissue after 1 h ischemia/4 h reperfusion. Asterisks indicate the endothelial lumen. Arrows indicate loss of syndecan‐1 in the endovascular lumen. (g) Wet‐to‐dry ratio of lung tissue after 1 h ischemia/4 h reperfusion. *p < 0.05, ***p < 0.001. No‐I/R, uninjured lungs; I/R, lungs after 1 h ischemia/4 h reperfusion; SF4, mice pretreated with HPSE inhibitor followed by I/R.

When Evans blue dye (EBD) accumulation was examined grossly as a measure of endothelial permeability, the lungs of WT I/R mice consistently contained more EBD after 1 h of ischemia followed by 4 h of reperfusion than WT uninjured lungs. Less EBD accumulated in the lungs of *HPSE* ^−/−^ mice and SF4‐treated WT mice after I/R injury (Figure 2a). Consistent with this gross, visual evaluation, significantly more EBD was extracted from the I/R‐injured lungs of WT mice than from uninjured WT lungs. Significantly less EBD was extractable from the lungs of SF4‐treated WT mice or *HPSE* ^−/−^ mice after I/R injury as compared with WT lungs with I/R injury (Figure 2b).

Plasma GAGs increased 6.2‐fold in the WT mice after I/R injury as compared with plasma GAGs in uninjured WT mice, indicative of glycocalyx degradation. SF4‐treated WT mice had significantly lower plasma GAG levels after I/R injury as compared with WT mice, and *HPSE* ^−/−^ mice did not show any increase in plasma GAGs after I/R (Figure 2c).

To further investigate the integrity of endothelial cells in the lungs after I/R injury, we examined caveolin‐1 expression in lung tissue by western blotting and found that caveolin‐1 protein was well preserved in the lung tissue after I/R in the WT mice treated with SF4 as compared with the WT I/R control mice. Additionally, caveolin‐1 was expressed at similar levels in the injured and uninjured lungs of *HPSE* ^−/−^ mice (Figure 2d). Consistently, the quantitative analysis of western blotting showed the expression of caveorlin‐1 after I/R in the WT mice treated with SF4 was significantly improved compared to that in the WT I/R control mice, while no significant difference was observed in lungs of *HPSE* ^−/−^ mice before and after I/R (Figure 2e). Immunofluorescent staining demonstrated that the glycoprotein syndecan‐1 disappeared from the microvascular endothelial lumen in the lungs of WT mice after I/R injury. In contrast, the lungs in SF4‐treated WT mice and HPSE^−/−^ mice retained syndecan‐1 in the microvascular lumen after I/R (Figure 2f).

To support these data, the wet‐to‐dry ratio of the lungs (W/D) was quantitated for each group and W/D in the lungs after I/R in the control WT mice was significantly increased compared to that of normal WT lungs. SF4 treatment reduced W/D but did not reach a statistical significance. There was no significant difference in W/D between the lungs with and without I/R injury in HPSE^−/−^ mice (Figure 2g).

### 3.3 Deleting the HPSE gene interferes with the gene expression and activities of other glycocalyx‐degrading enzymes

**FIGURE 3:**
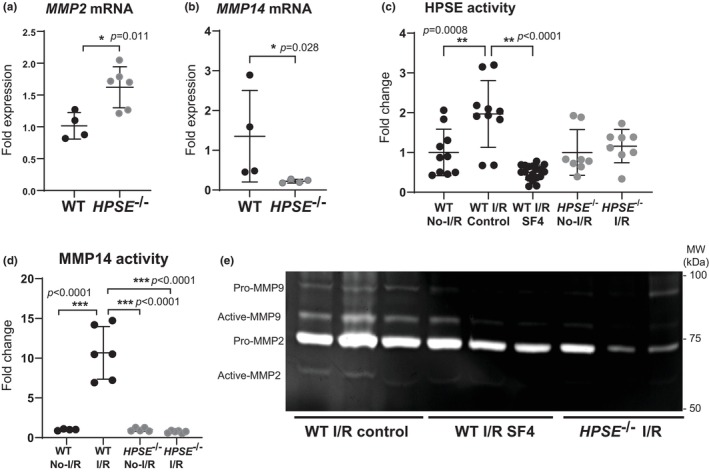
Gene expression and activity of glycocalyx‐degrading enzymes in lungs. Real‐time RT PCR was performed to determine the baseline mRNA expression levels of (a) matrix metalloproteinase (MMP) 2 and (b) MMP14 in lung tissue of WT and *HPSE* ^−/−^ mice. (c) HPSE activity and (d) MMP14 activity were determined in the lung tissue after I/R injury and in uninjured tissue and was normalized to the normal plasma level in each genotype WT or *HPSE* ^−/−^ to calculate the fold change. (e) Gelatin zymography was performed to visualize MMP2 and MMP9 activity in lung tissue after I/R injury. *p < 0.05, **p < 0.01, ***p < 0.001.

To examine if *HPSE* gene deletion affected other glycocalyx‐degrading enzymes, we first examined baseline mRNA expression for the matrix metalloproteases (MMPs) in the absence of lung I/R injury. *MMP2* mRNA expression was significantly higher in the lung tissue of *HPSE* ^−/−^ mice, while the *MMP14* mRNA expression was significantly decreased as compared with the lung tissue of WT mice (Figure 3a,b). Additionally, the baseline mRNAs for tissue inhibitors of MMPs (TIMP‐1, TIMP‐2, TIMP‐3, and TIMP‐4) and serpin‐1 (plasminogen activator inhibitor 1) were significantly downregulated in the lung tissue of *HPSE* ^−/−^ mice as compared with the lung tissue of WT mice (Figure S2). HPSE activity, measured using a β‐glucuronidase activity assay, was upregulated in the lungs after I/R injury in WT mice. This upregulation was significantly attenuated by SF4 treatment. In the lung tissue of *HPSE* ^−/−^ mice, HPSE activity was similar to that measured in uninjured WT mice and was unchanged after I/R injury (Figure 3c). MMP14 enzymatic activity increased in the lungs of WT mice after I/R injury as compared with uninjured lungs, but did not increase after I/R injury in the lungs of *HPSE* ^−/−^ mice (Figure 3d). Gelatin zymography demonstrated that the enzymatic activities of the active forms of MMP2 and 9 in the lungs after I/R injury were higher in the WT controls as compared with the SF4‐treated WT mice and the *HPSE* ^−/−^ mice. Unlike *MMP2* mRNA expression, overall MMP2 enzymatic activity, measured for both the pro and active forms, was lower in the lungs of *HPSE* ^−/−^ mice as compared with the lungs of WT mice after I/R injury (Figure 3e).

### 3.4 HPSE is an important enzyme for inflammatory cell infiltration into the lungs during I/R injury

**FIGURE 4:**
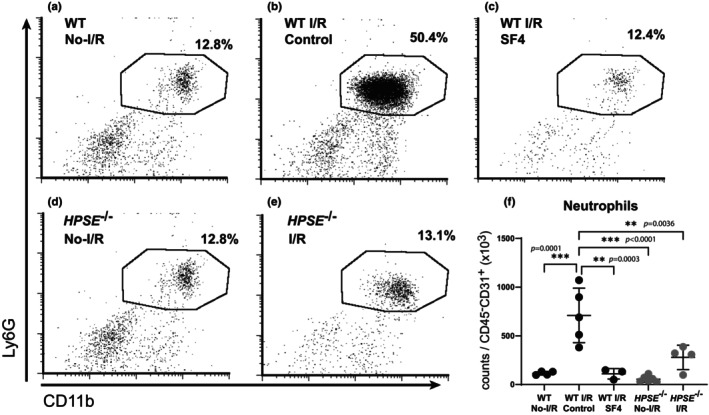
Flow cytometry analysis of infiltrating neutrophils and macrophages in lungs after I/R injury. The proportion of neutrophils (CD11b^+^ Ly6G^+^) in the CD45+ cell population in lung tissue from (a) WT uninjured lungs (No‐I/R), (b) WT lungs with I/R injury, (c) WT lungs pretreated with SF4 prior to I/R injury, (d) *HPSE* ^−/−^ uninjured lungs, and (e) *HPSE* ^−/−^ lungs subjected to I/R. (f) The cell counts for neutrophils are shown per 1000 endothelial cells (CD45^−^ CD31^+^) in the single‐cell suspension of the left lungs after I/R injury. **p < 0.01, ***p < 0.001.

Infiltration of inflammatory cells is one of the hallmarks of I/R injury; therefore, we used flow cytometry to investigate the cellular population of the lungs before and after I/R injury (Figure S3). The proportion of neutrophils (CD45^+^ CD11b^+^ Ly6G^+^) and the number of neutrophils counted in the single cell suspension were both increased in the lungs of WT mice after I/R injury as compared with uninjured WT lungs (Figure 4a,b,f and Figure S4a). Similarly, the counts of CD45^+^ CD11b^+^ CD11c^−^ SiglecF^−^ CD86^+^ population (possible M1 macrophages) were significantly increased in the lungs of WT mice after I/R injury as compared with uninjured WT lungs, while the counts of CD45^+^ CD11b^+^ CD11c^−^ SiglecF^−^ CD86^−^ population (possible M2 macrophages) were not significantly increased in the WT lungs after I/R injury (Figure S4b,c). Consistent with our other findings, SF4 treatment significantly decreased the number of neutrophils, M1 macrophages, and M2 macrophages in the lungs after I/R injury (Figure 4c,f–h). I/R injury did not lead to inflammatory cell infiltration into the lungs of *HPSE* ^−/−^ mice as the number of neutrophils, M1 macrophages, and M2 macrophages detected by flow cytometry did not increase (Figure 4d–h).

## 4 DISCUSSION

This study demonstrated that HPSE plays a key role in the development of endothelial dysfunction after lung I/R injury using a specific inhibitor and a genetic knockout mouse model. HPSE inhibition or deletion blocked the upregulation of pro‐inflammatory mediators during I/R, decreased or eliminated tissue infiltration by neutrophils and M1 macrophages after I/R, and minimized activation of MMPs in response to I/R. Tissue and endothelial integrity were better maintained after I/R injury when HPSE was absent or inhibited. Plasma GAG analysis and immunostaining for glycocalyx components revealed that HPSE is necessary for glycocalyx degradation in response to the I/R injury.

The glycocalyx is degraded enzymatically, and enzymes responsible for degradation have been identified for each component of the glycocalyx layer: HPSE for heparan sulfate, MMPs for proteoglycan core proteins, and hyaluronidase for hyaluronan. (Li et al., 2020) HPSE and the MMPs likely have proinflammatory properties, while hyaluronidase and heparinase‐2 may attenuate inflammatory responses. (Dokoshi et al., 2018; Parks et al., 2004; Petrey & de la Motte, 2014; Vlodavsky et al., 2020) Some reports have shown that the expression and activity of HPSE are a driving force in acute organ failure. (Goldberg et al., 2014; Schmidt et al., 2012) Consistent with these reports, we found that I/R injury increased the activity of HPSE and MMP2 in WT lungs, and that treatment with HPSE inhibitor or HPSE deletion attenuated the phenotype of I/R injury and preserved endothelial cells in the lungs. Interestingly, inhibition of MMP2 activation occurred following HPSE inhibition or when HPSE was deleted, suggesting a possible link between the HPSE activity and MMP2 activation. HPSE knockout mice have been studied previously, and HPSE was found to influence *MMPs* mRNA expression in the liver, kidneys and mammary glands. (Zcharia et al., 2009) Similarly, we observed the upregulation of MMP2 and the downregulation of MMP14 and TIMPs in uninjured *HPSE* ^−/−^ lungs at baseline. MMP2 is cleaved and activated by MMP14 and CD44 glycoprotein (Itoh & Seiki, 2006; van Hinsbergh & Koolwijk, 2007). MMP14 activity was increased in WT lungs after I/R injury but not in *HPSE* ^−/−^ lungs, implying a role for HPSE in MMP regulation. Indeed, the CD44 isoforms differ in function, which may be defined by heparan sulfate modifications, and co‐expression of HPSE and CD44 has been reported. (Jones et al., 2000; Kuniyasu et al., 2002; Nedvetzki et al., 2003) In addition, we investigated caveolin‐1 as an endothelial integrity marker and found its degradation in WT lungs after I/R injury but not in HPSE^−/−^ or SF4‐treated lungs, which was consistent with MMP2 activity. Caveolin‐1 has a primary role to keep caveolae function and structure on the endothelial surface and its regulatory role of MMP14 has been reported previously. (Kruglikov et al., 2020) Our findings suggest a potential interaction of HPSE on the caveolin‐MMP14 axis to activate MMP2 as well. Further study may be of interest to clarify the molecular mechanisms underlying this potential regulatory function of HPSE on MMP2 activation through caveolin‐1, MMP14 and CD44.

Cellular infiltration is a pathogenetic factor leading to tissue damage and inflammation during I/R injury. Neutrophils augment tissue inflammation during acute organ injury, and it has been previously reported that the activation of HPSE enhances neutrophil infiltration. (Khamaysi et al., 2017) HPSE from the neutrophils can degrade the extracellular matrix as well. (Bar‐Ner et al., 1987; Matzner et al., 1992) In this study, we found that HPSE inhibition or deletion prevented the infiltration of neutrophils into the lungs during I/R injury. Additionally, neutrophils are a known MMP9‐releasing cell population and require MMP9 to migrate to the respiratory tract. (Bradley et al., 2012) This study showed the MMP9 activity in lung tissue was significantly decreased after HPSE inhibition or deletion, which also indicated less neutrophil infiltration after I/R injury. Another important parameter contributing to inflammation is macrophage polarization to an inflammatory (M1) or regenerative (M2) phenotype. Previous studies demonstrated that HPSE may regulate macrophage polarization into classically activated M1 macrophages. (Gutter‐Kapon et al., 2016; Hermano et al., 2014; Masola et al., 2018) Supporting those findings, our study found that I/R injury significantly increased the M1 phenotype but not the M2 phenotype in the lungs after I/R injury. This promotion of the M1 phenotype was significantly decreased in the HPSE inhibitor‐treated or *HPSE* ^−/−^ lungs, suggesting that HPSE regulates the macrophage phenotypic changes during I/R injury.

This study has some limitations. Because we inhibited or eliminated HPSE systemically in mice in vivo, we could not investigate the role of HPSE derived from specific cells and tissues. Further experiments using in vitro or tissue/cell‐specific in vivo knockout models are needed to figure out the contribution and dominancy of HPSE derived from different tissue or cell types to the development of I/R injury. Also, this study used a hilar clamp model of lung I/R injury to examine acute outcomes. The model does not fully simulate transplant‐related I/R injury (e.g., cold ischemic injury) (Lama et al., 2017). A mouse transplant model should be employed in follow‐up studies to demonstrate the long‐term effects of HPSE inhibition or deletion on lung transplant outcomes. Finally, we did not use the vital dye to remove the dead cell population in the single cell suspension from the flow cytometry analysis, while we confirmed the cell viability by trypan blue before surface staining. These potential dead cells may affect our results (e.g., misinterpretation).

## 5 CONCLUSION

HPSE is a potential therapeutic target to attenuate graft damage in lung transplantation. HPSE inhibition may be a practical strategy to preserve endothelial cells in the lungs and attenuate I/R injury, leading to better posttransplant outcomes.

## AUTHOR CONTRIBUTIONS

All authors reviewed and approved the final manuscript. *Participated in research design*: Kentaro Noda and Pablo G. Sanchez. *Participated in the performance of the research*: Kentaro Noda, Neha Atale, Amer Al‐Zahrani, Masashi Furukawa, Mark E. Snyder, Xi Ren, and Pablo G. Sanchez. *Participated in the data analysis*: Kentaro Noda, Neha Atale, Amer Al‐Zahrani, Masashi Furukawa, Mark E. Snyder, Xi Ren, and Pablo G. Sanchez. *Participated in the writing of the paper*: Kentaro Noda, Neha Atale, Amer Al‐Zahrani, Masashi Furukawa, Mark E. Snyder, Xi Ren, and Pablo G. Sanchez.

## FUNDING INFORMATION

This study was supported by the Vascular Medicine Institute, the Hemophilia Center of Western Pennsylvania, and Vitalant (P3HVB) and by the Department of Cardiothoracic Surgery at the University of Pittsburgh.

## CONFLICT OF INTEREST STATEMENT

All authors declare no conflict of interests.

## Supporting information


**Data S1:** Supporting information.

## Data Availability

All data associated with this study are present in the main text or the Supplementary Materials.
