# Supplementary material for: Heparanase‐induced endothelial glycocalyx degradation exacerbates lung ischemia/reperfusion injury in male mice
Source: Physiol Rep. 2024 Oct 24;12(20):e70113. doi: 10.14814/phy2.70113 (PMC11502304; doi:10.14814/phy2.70113)
Supplement: Supplementary file 1 — Data S1: Supporting information. [file PHY2-12-e70113-s001.docx]

**The role of heparanase during ischemia/ reperfusion injury in the lungs of male mice**

Noda K et al, University of Pittsburgh

**Table for context**

| **Page** | **Context** |
| --- | --- |
| 2 | Figures S1 Supplemental data for figure 1H, western blotting for I-κBα. |
| 3 | Figure S2. The effect of *HPSE* gene deletion on the expression of other glycocalyx-regulating enzymes. |
| 3 | Figure S3. Strategy employed for the flow cytometry analysis. |
| 4 | Figure S4. Supplemental data for flow cytometry data |

**Supplemental Figures**


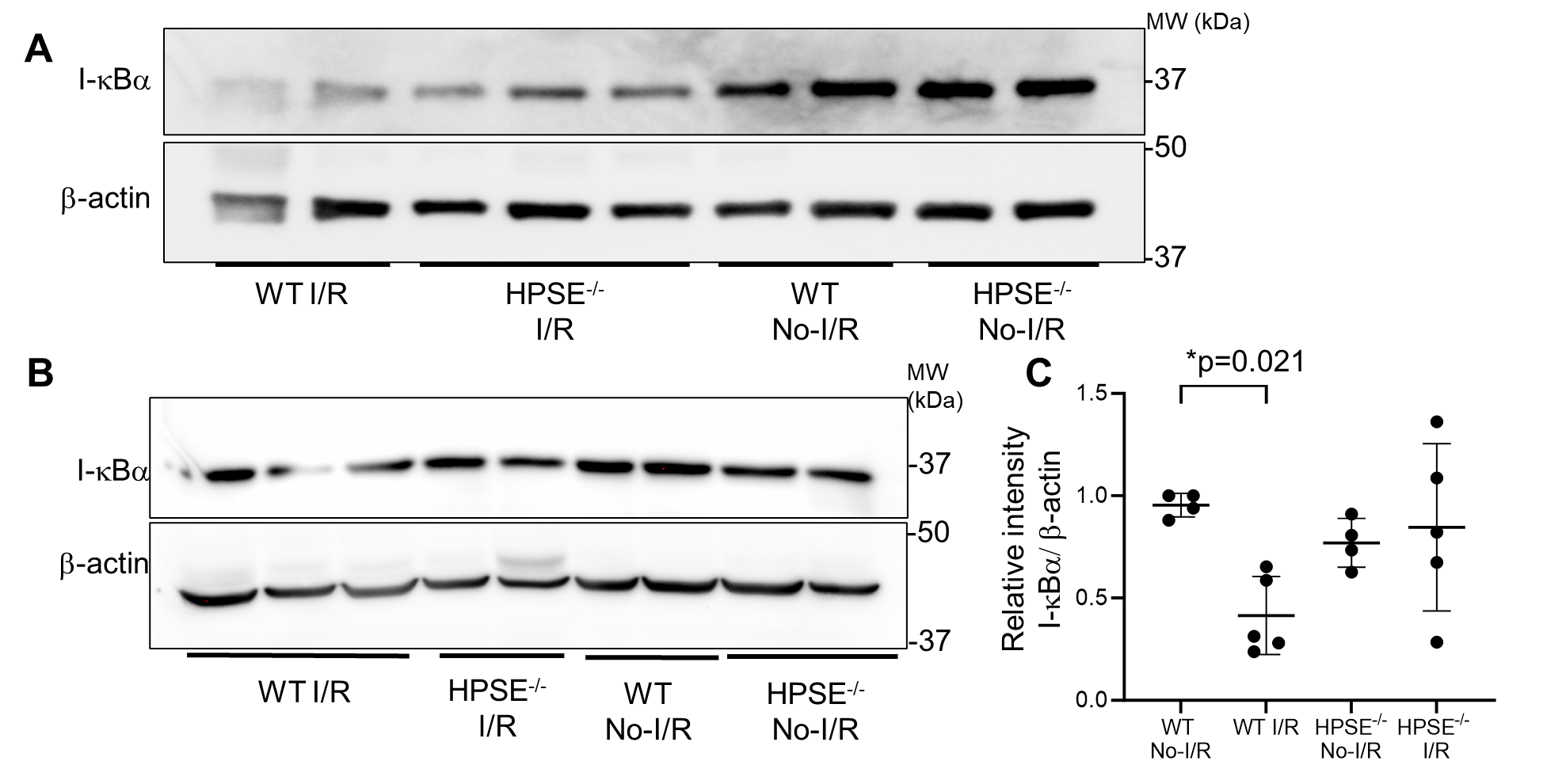


**Figure S1. Supplemental data for figure 1H, western blotting for I-κBα.** **(A,B)** The representative images of western blotting for inhibitor of nuclear factor κB α (I−κBα) and β-actin expression. (C) Quantitative data of relative intensity of each band of I−κBα to β-actin. *p<0.05.





**Figure S2. The effect of *HPSE* gene deletion on the expression of other glycocalyx-regulating enzymes.**

Real-time RT PCR was performed to quantify the mRNA expression of (A) Serpin-1, (B) TIMP-1, (C) TIMP-2, (D) TIMP3, and (E) TIMP-4 in WT and *HPSE*^-/-^ lungs. White bars, WT; gray bars, *HPSE*^-/-^. TIMP, tissue inhibitor of matrix metalloproteinase.
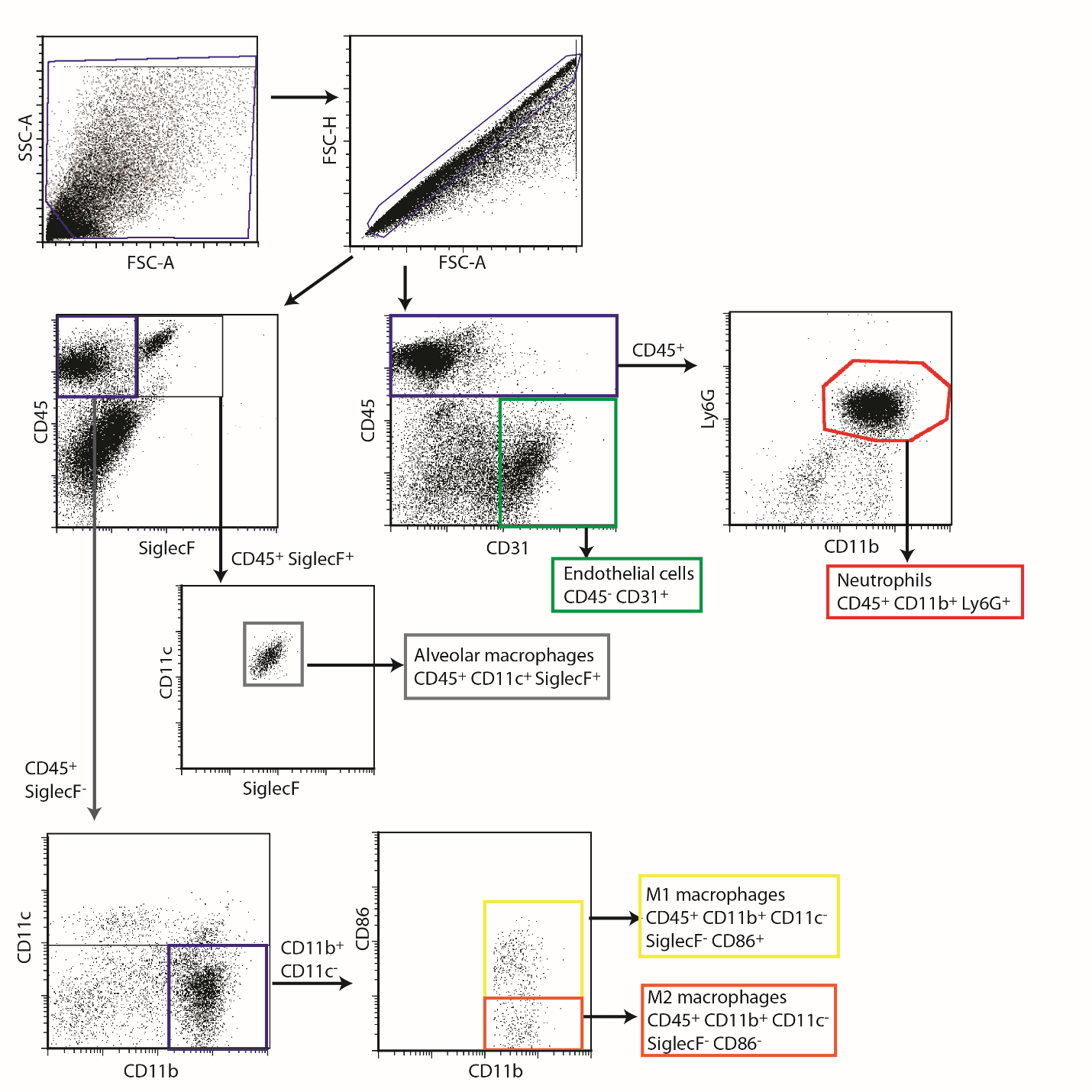


**Figure S3. Strategy employed for the flow cytometry analysis.**

Representative flow-cytometric plots of flow cytometry data of single-cell suspensions from mouse lungs and the gating strategy used to identify endothelial cells, neutrophils, alveolar/M1/M2 macrophages. FSC, forward scatter; SSC, side scatter.


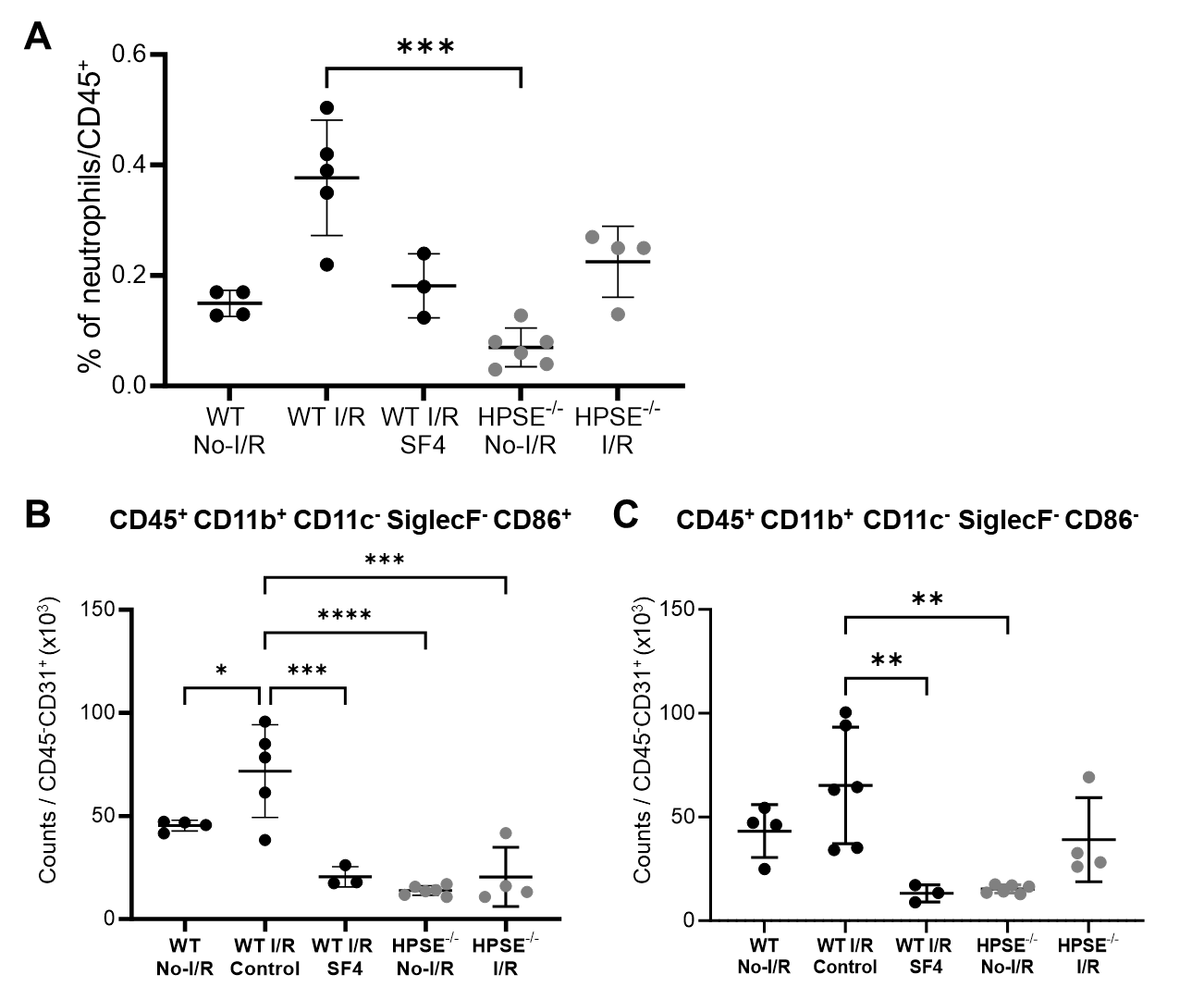


**Figure S4.** **Supplemental data for flow cytometry data. (A)** the percentage of neutrophils in the CD45^+^ population. (B, C) The cellular count for CD45^+^ CD11b^+^ CD11c^-^ SiglecF^-^ CD86^+^ population and CD45^+^ CD11b^+^ CD11c^-^ SiglecF^-^ CD86^-^ population in single cell suspension of lungs after I/R injury were normalized by endothelial cells x 10^3^ (CD45^-^ CD31^+^). ***p<0.001, **p<0.01, *p<0.05.
